# Application of Hybrid Electrically Conductive Hydrogels Promotes Peripheral Nerve Regeneration

**DOI:** 10.3390/gels8010041

**Published:** 2022-01-06

**Authors:** Fengshi Zhang, Meng Zhang, Songyang Liu, Ci Li, Zhentao Ding, Teng Wan, Peixun Zhang

**Affiliations:** 1 Department of Orthopedics and Trauma, Peking University People’s Hospital, Beijing 100044, China; xmx066@pku.edu.cn (F.Z.); mengzh2008@bjmu.edu.cn (M.Z.); 1911110343@pku.edu.cn (S.L.); drlici@bjmu.edu.cn (C.L.); zhentao_ding@stu.pku.edu.cn (Z.D.); tengwan.med@hotmail.com (T.W.); 2 Key Laboratory of Trauma and Neural Regeneration, Ministry of Education, Beijing 100044, China; 3 National Center for Trauma Medicine, Beijing 100044, China

**Keywords:** conductive hydrogel, tissue engineering, peripheral nerve regeneration, cell proliferation

## Abstract

Peripheral nerve injury (PNI) occurs frequently, and the prognosis is unsatisfactory. As the gold standard of treatment, autologous nerve grafting has several disadvantages, such as lack of donors and complications. The use of functional biomaterials to simulate the natural microenvironment of the nervous system and the combination of different biomaterials are considered to be encouraging alternative methods for effective tissue regeneration and functional restoration of injured nerves. Considering the inherent presence of an electric field in the nervous system, electrically conductive biomaterials have been used to promote nerve regeneration. Due to their singular physical properties, hydrogels can provide a three-dimensional hydrated network that can be integrated into diverse sizes and shapes and stimulate the natural functions of nerve tissue. Therefore, conductive hydrogels have become the most effective biological material to simulate human nervous tissue’s biological and electrical characteristics. The principal merits of conductive hydrogels include their physical properties and their electrical peculiarities sufficient to effectively transmit electrical signals to cells. This review summarizes the recent applications of conductive hydrogels to enhance peripheral nerve regeneration.

## 1. Introduction

Peripheral nerve injury (PNI) is mainly caused by trauma and surgery [1,2]. Although the peripheral nervous system (PNS) has the intrinsic capacity for spontaneous regeneration and axon regrowth to a certain extent, its regenerative capacity is limited [3,4]. The mechanism of nerve regeneration is complex, the speed of nerve growth is relatively slow, and the target muscle loses innervation and then atrophies [5,6]. Persistent sensory and motor defects are common in the affected nerve control areas, which may develop neuropathic pain and cause lifelong disability due to limb paralysis [7]. This causes a decline in the quality of life and psychological obstacles to patients and brings a significant economic and social burden. Therefore, it is necessary to use a variety of positive approaches to enhance peripheral nerve regeneration and reestablish synaptic connections with target organs as soon as possible to avoid dysfunction caused by denervation.

After PNI, distal injured nerve fibers experience Wallerian degeneration [8,9,10]. Denervated Schwann cells (SCs) proliferate, lengthen, and rearrange to direct and facilitate axonal regeneration. Axons arise from living nerve stumps attached to neuronal bodies [11,12,13]. The myelin-associated genes of SCs are down-regulated, while the growth-associated genes of SCs and neurons are up-regulated [14,15,16]. Nevertheless, the change in gene expression is momentary and gradually fails to support axonal regeneration in endoneurial tubes [17,18]. Presently, end-to-end neurorrhaphy is the common treatment for peripheral nerve damage smaller than 1 cm, while autologous nerve grafts are considered the clinical gold standard for nerve defects larger than 1 cm [19]. However, there are still key issues limiting the use of nerve grafts, such as the lack of donor nerve tissues, multiple operations, neuroma formation, donor site morbidity, and possible immunological responses [20,21].

In view of the shortcomings of the current methods for the treatment of PNI, many researchers have been devoting themselves to developing novel strategies as potential therapeutic methods for peripheral nerve regeneration [19]. Neural tissue engineering combines the principles and techniques of neurobiology, engineering, and material science and imitates natural nerve tissue structure and physiological characteristics [22,23,24]. The fundamental purpose of designing and manufacturing nerve tissue substitutes that simulate the three-dimensional microstructure and mechanical properties of the complex extracellular matrix (ECM) microenvironment is to regenerate the functional properties of damaged nerve tissue [25,26]. Therefore, multifunctional nerve tissue substitutes with biological, chemical, and physical cues and simulating the cellular microenvironment play an essential role in successfully controlling neuronal cell growth, proliferation, directional migration, differentiation, and nerve tissue regeneration [27,28].

Studies have shown that neuronal cells can transmit electrical signals along axons, and the electric field plays a positive role in cellular alignment, proliferation, migration, differentiation, paracrine activity, and structural and functional recovery after PNI [29,30]. Therefore, the designed nerve tissue substitutes should have electrical conductivity to simulate the characteristics of the ECM and then regulate the physiological activities of cells and nerve regeneration by electric field stimulation [31,32].

In addition to electrical conductivity, nerve tissue substitutes should also have certain characteristics, such as a three-dimensional porous structure, better mechanical properties, and topographic/physical cues to provide a better simulation of the natural ECM [33,34]. Hydrogel is a very attractive biomaterial. Hydrogels have a three-dimensional crosslinking network composed of hydrophilic organic polymers, which can absorb a large amount of water, their morphology is soft and elastic, and irritation to biological tissue can be minimized [35,36]. Hydrogels can maintain their inherent three-dimensional porous structure, making them suitable for cell processes in vivo applications, including adhesion, proliferation, and migration, and favor the transportation and retention of nutrients and growth factors [37,38]. In addition to the above advantages, hydrogels also have good biocompatibility, biodegradability, and low immunogenicity [39,40]. Therefore, hydrogels can provide a suitable microenvironment for peripheral nerve regeneration and make them new biomaterials with wide application prospects in neural tissue engineering [41,42].

**Figure 1:**
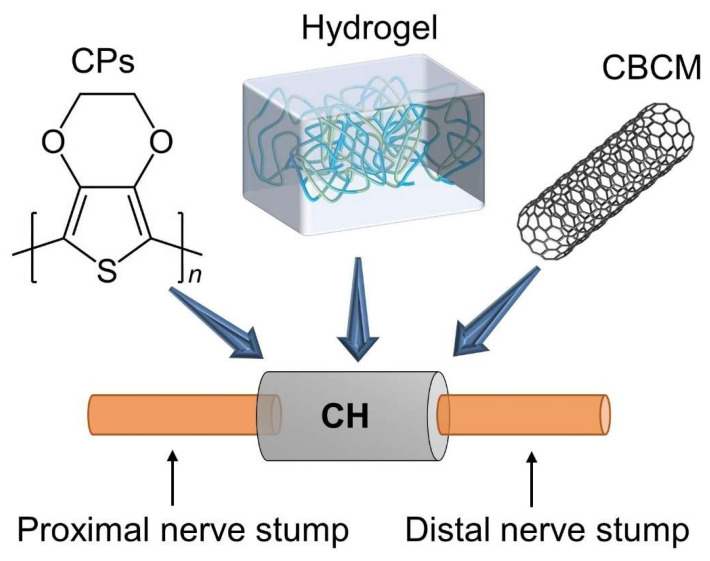
Schematic of conductive hydrogels (CH) for peripheral nerve regeneration. CPs = conductive polymers; CBCM = carbon-based conductive materials; CH = conductive hydrogels.

Over the past few decades, the growing demand for neural tissue engineering has led to innovative synthetic strategies to produce safer and more efficient biomaterials for PNI repair. In this context, conductive hydrogels (CHs) have attracted great attention from researchers. CHs are composed of conductive materials and hydrogels, which not only maintain their inherent conductivity but also have the excellent properties of hydrogels, such as elevated water composition, porosity, softness, plasticity, mechanical behavior, and large surface area, which promote the application of CHs in tissue engineering [43,44,45]. CHs have a suitable three-dimensional microstructure and mechanical properties and can also replicate the biological and electrical properties of biological tissues that need to conduct electricity and realize cell manipulation based on electrical signals to promote the proliferation and differentiation of neurons. CHs are amongst the most effective biomaterials to simulate human tissues’ biological and electrical behavior (Figure 1).

In this review, we summarized the applications of various types of CHs in peripheral nerve regeneration, discussed the biological characteristics of CHs, and proposed challenges and possible future development directions in the application of CH in nerve repair.

## 2. Conductive Hydrogels Applied in Peripheral Nerve Injury

CHs have excellent biocompatibility and adjustable conductivity and are easy to synthesize and modify [46]. By promoting signal transduction between cells, CHs can enhance the proliferation and differentiation of nerve cells, which is beneficial to the repair and regeneration of nerve tissue—in addition, mimicking intricate tissue architecture and essential cellular microenvironments are critical parameters when fabricating CHs (Table 1).

### 2.1. Conductive Polymers (CPs)-Incorporated CH

**Figure 2:**
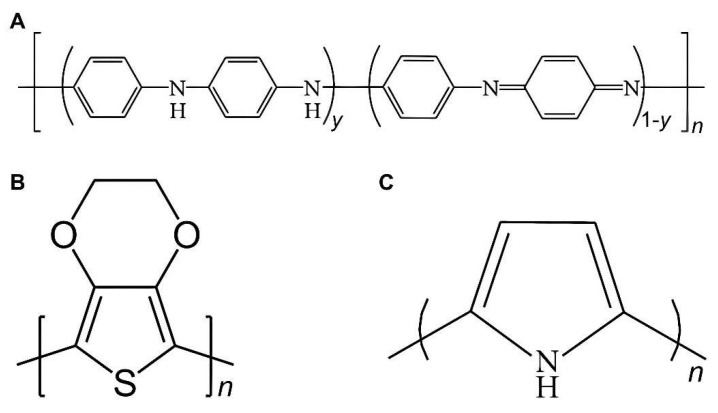
New generation of conductive polymers (CPs). (**A**) polyaniline (PANi); (**B**) poly(3,4-ethylenedioxythiophene) (PEDOT); (**C**) polypyrrole (PPy).

A new generation of conductive polymers (CPs), such as polypyrrole (PPy), poly(3,4-ethylenedioxythiophene) (PEDOT), and polyaniline (PANi) (Figure 2), have not only electrical conductivity similar to metal and inorganic semiconductors but also have good biocompatibility and are easy synthesize [46]. Studies have shown that CPs can enhance cell activity, promote cell adhesion, differentiation, migration, and proliferation, and facilitate cell secretion function at the material-tissue interface [59,60]. When CPs exist in animals for a long time, they have no obvious adverse effect on animals or only produce weak inflammatory reactions. Therefore, CPs are very suitable for electrical signal-sensitive tissue repairs, such as skin, nerve, myocardium, skeletal muscle, and bone [46,61,62,63]. Based on CPs, researchers further developed CHs, which are more compatible and adjustable to nerve tissues and promote peripheral nerve regeneration.

#### 2.1.1. PEDOT-Incorporated CH

Yamamoto et al. prepared thermostable and electric-conducting poly(2,5-thienylene) (PTH) [64]. Although PTH has good electrical conductivity, its processability is poor, limiting its application. Based on PTH, PEDOT with excellent electrical activity and chemical stability was further prepared [64]. Studies have shown that PEDOT has good biocompatibility with both cells and tissues [65].

Abidian et al. provided a novel hybrid conduit made up of electrically polymerized PEDOT and agarose hydrogel. By electrodepositing two layers of PEDOT, the PEDOT-modified agarose conduits were mechanically reinforced and further conductive. Then, the conduits were implanted to repair 10 mm peroneal nerve gaps of rats. At 12 weeks after the operation, the extensor digitorum longus (EDL) muscle mass, EDL maximal specific muscle force and peroneal nerve histomorphometry were measured to evaluate the effects of the nerve gap conduits. Their results indicated that PEDOT-modified agarose conduits provided significantly higher EDL muscle mass, EDL maximal specific muscle force, myelinated axon number, nerve fiber diameter, axon diameter, and myelin thickness than plain agarose conduits [47].

**Figure 3:**
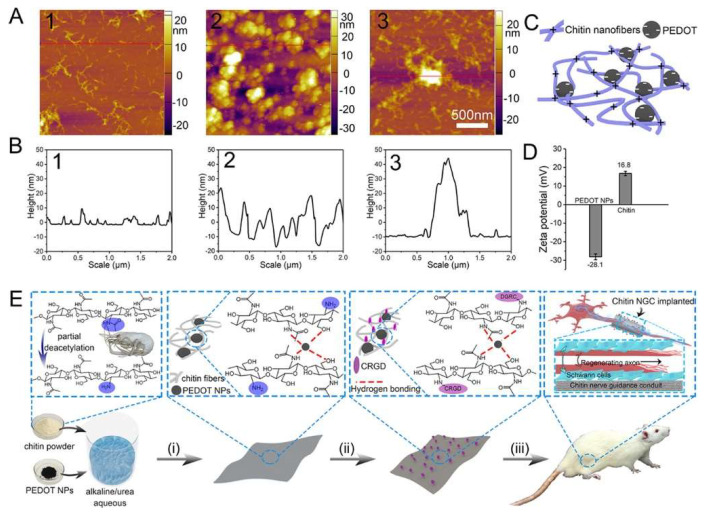
AFM images of the diluted partial deacetylation chitin solution, PEDOT nanoparticles, and chitin blended with PEDOT nanoparticles solution ((**A**), 1–3) and the corresponding height graphs ((**B**), 1–3). Schematic model of chitin/PEDOT solution (**C**), and ζ–potential of PEDOT NPs and chitin (**D**). Illustration for the preparation of conductive chitin hydrogel used in peripheral nerve regeneration (**E**): (i) preparation for partial deacetylation of chitin blended with PEDOT NP hydrogel film (ChT–PEDOT); (ii) modification of the cell adhesive peptide CGRD onto the chitin hydrogel film surface (ChT–PEDOT–p); and (iii) implantation of ChT–PEDOT–p in sciatic nerve defect rat to evaluate the recovery ability. (For interpretation of the references to color in this figure legend, the reader is referred to the web version of this article.) This figure was published in [48]—copyright, American Chemical Society (2021). Permission to share the material has been granted.

Huang et al. used polysaccharide chitin to construct a hydrogel film to direct the regeneration of injured sciatic nerves by integrating PEDOT nanoparticles (NPs) and the cell adhesive peptide Cys-Arg-Gly-Asp (CRGD). In the process of chitin partial deacetylation, the electrostatic interaction between the negatively charged PEDOT NPs and the chitin (amino groups) was enhanced, leading to the improved mechanical performance of the chitin/PEDOT hydrogel (Figure 3). Because of the optimized properties, such as the porous structure and biocompatibility, the hydrogel significantly enhanced RSC-96 cell proliferation and adhesion. The expression of Schwann cell activity-related genes, including S100, myelin basic protein (MBP), and NF-200, was also promoted. In the repair evaluation of 10 mm rat sciatic nerve defects, the chitin/PEDOT hydrogel efficiently promoted nerve regeneration. Compared to the autograft group, the thickness of the regenerated myelin, compound muscle action potential (CMAP), the average diameter of muscle fibers, and wet weight ratios of gastrocnemius in the chitin/PEDOT hydrogel group exhibited similarity. The evaluation of the regenerated nerve through immunohistochemistry, immunoblotting, and immunofluorescence showed that angiogenesis and Schwann cell adhesion and proliferation were promoted. The molecular mechanism of angiogenesis was further identified using western blotting. The amounts of cell proliferation- and apoptosis-related AKT, activity of monocyte- and macrophage-related VEGFR1, and mammalian cell metabolism-related AMPKα in the chitin/PEDOT hydrogel group were significantly higher than those in the chitin group [48].

#### 2.1.2. PANi-Incorporated CHs

PANi is synthesized from aniline monomers by electrochemical or chemical oxidation polymerization and has excellent conductivity and good biocompatibility [66]. Studies have shown that composite conductive materials based on PANi can promote the proliferation and differentiation of neurons [67,68].

Xu et al. constructed PANi/cellulose composite hydrogels with hierarchical micronanostructures. Cellulose hydrogel was used as a template to synthesize PANi in situ. The polymer has one conductive side through the limited interfacial polymerizing method. In the presence of water, hierarchical micronanostructure formation was induced by the interactions between hydrophobic PANi, hydrophilic cellulose, and the phytic acid bridge. The cellulose hydrogel’s three-dimensional network structure can provide full cavities for PANi polymerization and a backbone protecting and immobilizing the micronanostructure. The submicrometer particles of PANi made up of PANi nanoparticles and nanofibers were evenly integrated into the cellulose matrix. The PANi/cellulose hydrogels had soft physical properties, excellent biocompatibility, and exceptional conductivity, which facilitated sciatic nerve regeneration in rats. Their results showed that pure cellulose was an inert substance in nerve repair, while the PANi of PANi/cellulose hydrogels played an essential role in regenerating peripheral nerves. The electrical conductivity and hierarchical micronanostructure of the conduits promoted the attachment and extension of neurons [49].

Dong et al. developed a tough CH by copolymerizing polyacrylamide (PAM) and PANi. This CH had good biocompatibility, excellent mechanical properties, and electrical conductivity which were similar to those of natural nerve tissues. By means of near-infrared light, PANi enhanced the bioelectrical signals, which helped repair damaged peripheral nerves. This CH still had high electrical conductivity durability after being mechanically elongated. Therefore, it could adapt to unexpected nerve tissue tension during motion. This CH successfully replaced the damaged sciatic nerve of the toad in vitro. Moreover, in vivo results demonstrated that this CH could replace the loss of sciatic nerves in rats as a highly conductive bridge [49].

#### 2.1.3. PPy-Incorporated CHs

PPy is synthesized by electrochemical or chemical oxidation polymerization of pyrrole monomers [69]. PPy has good electrical conductivity and excellent chemical stability, so it is widely used in the field of biomedicine [49]. Studies have shown that PPy has good biocompatibility with cells and tissues, and the body will not produce obvious inflammatory reactions after being implanted in the body for a long time [70,71].

Bu et al. developed a straightforward method to fabricate conductive sodium alginate (SA) and carboxymethyl chitosan (CMCS) hydrogels (SA/CMCS/PPy) with good mechanical and biocompatibility properties. With the presence of calcium ions from the sustained release system made up of D-glucono-d-lactone (GDL) and superfine calcium carbonate (CaCO_3_), SA/CMCS was crosslinked and PPy provided the electrical conductivity of this hydrogel. Meanwhile, PPy adjusted the porosity, swelling ratio, Young’s modulus, and gelation time of these conductive SA/CMCS/PPy hydrogels. The conductivity was from 2.41 × 10^−5^ to 8.03 × 10^−3^ S cm^−1^. The mechanical performance was excellent when the feed ratio of PPy was 0.20 while the mass ratio of SA:CMCS was 2:1. This SA/CMCS/PPy CH showed high biocompatibility for RSC96, PC12, and bone marrow mesenchymal stem cells (BMMSCs), and its ECM-simulated structure supplied suitable conditions for adhesion and proliferation of cells. The biocompatibility of this CH was confirmed using a subcutaneous inflammatory reaction assay. As the filling material in nerve guide conduits, this CH played a crucial role in providing great assistance for peripheral nerve regeneration [51].

**Figure 4:**
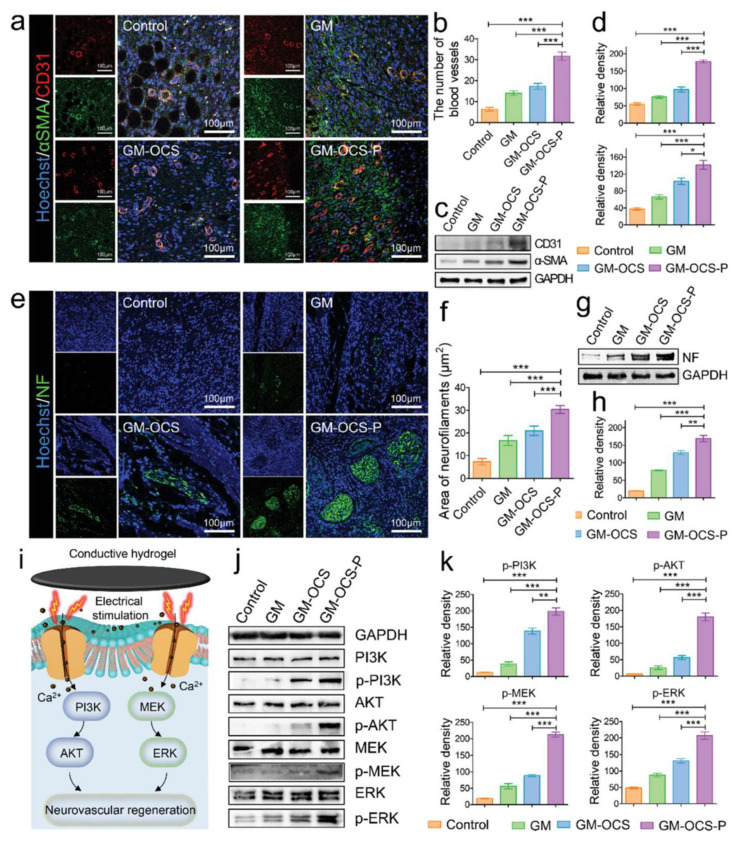
The mechanism of conductive hydrogel promoting neurovascular regeneration in vivo. (**a**) Images of immunofluorescence co-staining for CD31 and αSMA at day 14. (**b**) Quantitative analysis of the blood vessel number (*n* = 3). (**c**) Protein expressions of CD31 and αSMA at the diabetic wound site were measured by western blot assays. (**d**) Quantitative analysis of protein expression (*n* = 3). (**e**) Images of immunofluorescence for NF staining at day 14. (**f**) Quantitative analysis of the area of neurofilaments (*n* = 3). (**g**) Protein expressions of NF at the diabetic wound site were measured by western blot assays. (**h**) Quantitative analysis of protein expression (*n* = 3). (**i**) Schematic illustration of the mechanism conductive hydrogel uses to promote neurovascular regeneration. (**j**) Protein expressions of MEK, p-MEK, ERK, p-ERK, PI3K, p- PI3K, AKT, and p-AKT were evaluated by western blot. (**k**) Quantitative analysis of protein expression (*n* = 3). Statistical analysis was implemented by using One-way ANOVA with Bonferroni’s test (* *p* < 0.05, ** *p* < 0.01, *** *p* < 0.001). (For interpretation of the references to color in this figure legend, the reader is referred to the web version of this article.) This figure was published in [52]—copyright, John Wiley and Sons (2021). Permission to share the material has been granted.

Fan et al. fabricated an ECM-mimicked conductive dressing made up of an interpenetrating polymer network hydrogel consisting of gelatin methacryloyl (GelMA), oxidized chondroitin sulfate (OCS), and OCS-PPy electrically conductive nanoparticles. This CH had soft mechanical properties, good electrochemical performance, a three-dimensional porous structure, and excellent adhesiveness, providing the tissue-matching conductivity and mechanical conditions required for the regeneration of neurovascular tissues. In vitro and in vivo studies indicated that this CH had good biocompatibility and promoted nerve cell migration, axon elongation, and angiogenesis by increasing the intracellular Ca^2+^ concentration. The increased Ca^2+^ concentration enhanced protein phosphorylation in the phosphatidylinositol 3-kinase (PI3K)/protein kinase B (AKT) and mitogen-activated protein kinase kinase (MEK)/extracellular signal-regulated kinase (ERK) pathways (Figure 4) [52].

Liu et al. developed biocompatible CH with soft, porous, and adhesive properties. With the effect of an oxidative initiator (FeCl_3_), the hydrogel was constructed by gelation because of the crosslinking of PPy and tannic acid (TA). Due to good self-healing and adhesive properties, these thin film-like hydrogels could be attached easily to the damaged nerves. Afterward, it automatically warped a tubular structure without unnecessary invasive operation. This hydrogel provided a stable and appropriate bridge connection for the nerve tissues. In vitro results showed that the hydrogels facilitated SC adhesion and migration and promoted axonal extension. In vivo studies showed that this CH stimulated regeneration and remyelination of axons in diabetes mellitus rats. Besides, this hydrogel promoted nerve impulse conduction as well as muscle receptivity. As a result, it could prevent denervation atrophy of muscles and promote functional recovery [53].

### 2.2. Carbon-Based Conductive Materials (CBCM)-Incorporated CHs

Carbon-based conductive materials (CBCM), including graphene and carbon nanotubes (CNT) can also be integrated into nonconductive biomaterials to supply structural reinforcement and provide new advantages, including exceptional electrical and thermal conductivity, chemical stability, and biocompatibility [72,73]. These materials can mediate cell adhesion, proliferation, and differentiation, making them well suited for nerve tissue repair.

#### 2.2.1. CNT-Incorporated CH

CNT can be prepared by chemical vapor deposition, laser cutting, or arc discharge [74]. The nanoscale dimensions, low density, high aspect ratio, and electrical properties of CNT facilitate its application in biomedicine [74]. When utilized in the form of suspension, CNT can cause toxic responses by inducing oxidative stress in cells. Nevertheless, the toxic effect can be eliminated through surface functionalization or immobilizing CNT to a platform.

Koppes et al. selected single-walled CNT as a model nanofiller manipulating the electrical characteristics of collagen type I-10% Matrigel to fabricate an electrically conductive three-dimensional composite hydrogel. The single-walled CNT-loaded composite hydrogels resulted in greater conductivity without significant changes in the elastic modulus. The total neurite outgrowth and neurite persistence length of primary DRG encapsulated within the single-walled CNT loaded composite hydrogels were significantly enhanced compared to the nanofiller-free control. Furthermore, DRG outgrowth was stronger after combining exogenous electrical stimulation with this CH [54].

He et al. prepared a hybrid nanofibrous hydrogel with good injectability and conductivity by homogeneously integrating CNT into a functional self-assembling peptide (SAP). 2D (on the surface of hybrid hydrogel) and 3D (within the hydrogel) culture experiments showed that electrical stimulation could enhance axonal outgrowth and SC migration away from DRG [55].

#### 2.2.2. Graphene-Incorporated CH

Graphene can be prepared through mechanical exfoliation, liquid-phase exfoliation, and chemical vapor deposition [74]. Graphene oxide (GO) is obtained through the hybridization of carbon atoms, can be dispersed easily in water, and can interact with diverse inorganic or organic materials [75]. Nevertheless, the conductivity of GO-based materials is limited because of the existence of oxides. This faultiness can be optimized by reducing GO via laser or thermal processing. Reduced GO (rGO) has augmented electrical and physical properties [76]. Graphene-based materials facilitate physicochemical interactions to promote cellular attachment and proliferation and can be used to fabricate conductive and biocompatible materials [74].

**Figure 5:**
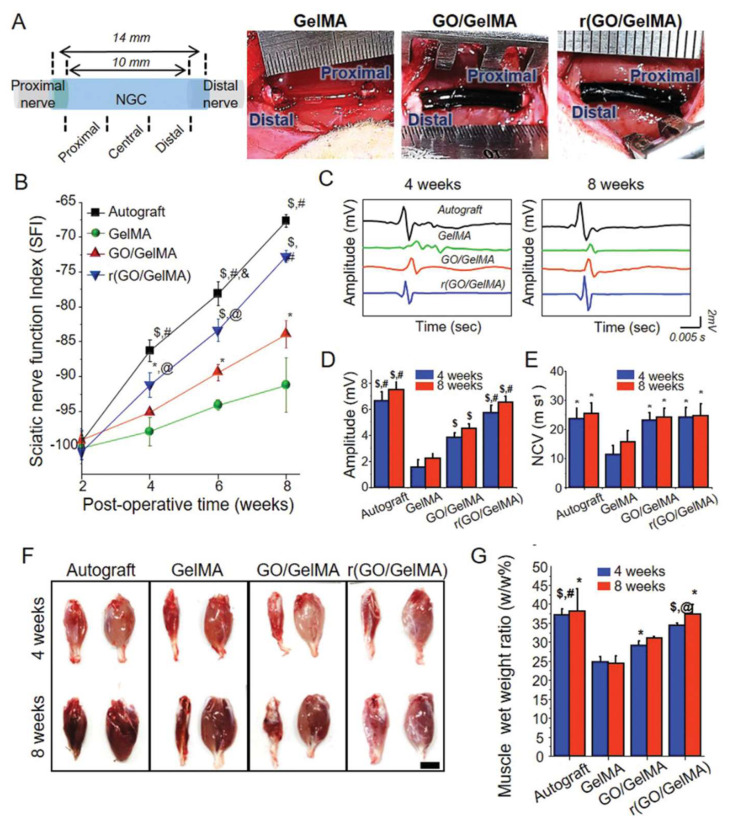
In vivo implantation of NGCs and functional recovery. (**A**) NGCs were implanted in a 10 mm gap of the sciatic nerve. (**B**) SFI of different groups at predetermined time points. (**C**) Comparison of electrophysiological recordings of compound muscle action potentials (CMAPs) for various implanted NGCs and autografts. (**D**) Onset–to–peak amplitude and (**E**) NCV of each group. (**F**) Images of muscles and (**G**) muscle wet weight ratio in each group. Scale bar: 25 mm. *, *p* < 0.05 compared to GelMA at the same week. $, *p* < 0.01 compared to GelMA at the same week. @, *p* < 0.05 compared to GO/GelMA at the same week. #, *p* < 0.01 compared to GO/GelMA at the same week. &, *p* < 0.05 compared to r(GO/GelMA) at the same week. (For interpretation of the references to color in this figure legend, the reader is referred to the web version of this article.) This figure was published in [56]—copyright, John Wiley and Sons (2020). Permission to share the material has been granted.

Park et al. fabricated a conductive reduced (GO/GelMA) (r(GO/GelMA)) hydrogel through the polymerization and subsequent chemical reduction of GO and GelMA. This multifunctional material had excellent flexibility, electrical conductivity, permeability, and mechanical stability, suitable for utilization as nerve conduits. In vitro results showed that, compared to GO-free GelMA, r(GO/GelMA) significantly improved PC12 proliferation and differentiation, likely because of the electroactivity and molecular interactions of rGO in the hydrogels. In vivo studies with a 10 mm sciatic nerve gap rat model demonstrated that nerve regrowth, remyelination, and functional recovery of muscles were significantly facilitated by r(GO/GelMA) conduits without toxicity (Figure 5) [56].

Peng et al. constructed a new self-adaptive all-in-one transmitting chip (GO:PPy:alginate-Chip) that combined therapeutic gene delivery, protein release, and electrical conduction into one microfluidic chip through three-dimensional coaxial printing. GO:PPy:alginate-Chip consisted of an inner microchannel full of enzyme-initiated plasmid DNA microcomplexes and an outer electrically conductive hydrogel shell decorated with chemokines. The chip delivered functional plasmid DNAs and chemokines and enhanced electrical conductivity via a self-adaptive procedure that markedly promoted endogenous mesenchymal stem cell recruitment and enhanced nerve regeneration [58].

Huang et al. engineered a conductive double-network (DN) hydrogel scaffold decorated with netrin-1 and supported by graphene mesh. Through the fast exchange of ions and ultraviolet irradiation, natural GelMA and alginate were entangled to form the hydrogel. This hydrogel could provide good biocompatibility and suitable mechanical strength and serve as a reservoir of netrin-1. Furthermore, the graphene mesh could enhance SC proliferation and guide their alignment. The scaffold had an acceptable Young’s modulus matching peripheral nerves and satisfactory electrical conductivity. Moreover, netrin-1 had double roles in inducing axon pathfinding and the migration of neurons. This netrin-1-laded graphene/DN hydrogel scaffold could markedly enhance peripheral nerve regeneration and the recovery of denervated muscles, which was even better than autologous grafts [58].

## 3. Challenges and Futures

Because of the high incidence and poor prognosis, PNI brings great pain to patients and brings a huge burden to the country and society, so it is a thorny problem perplexing global public health. Traditional treatment cannot meet the high requirements of PNI repair. As the gold standard, the application of nerve transplantation therapy is limited because of the shortage of donor nerves and the functional damage of donor nerve target organs. In this context, the strategy of using tissue engineering to repair PNI has gradually attracted the attention of researchers [77,78,79]. Several studies have proven that CHs have great advantages in repairing PNI. CHs are a promising application of neural tissue engineering and solving difficult clinical problems. However, there are still some unsolved challenges from the current research status, and plenty of work will need to be done.

First, CHs should be matched with complex human microenvironments to ensure safety. However, CH may be distributed and deposited in cells and organs, resulting in toxic reactions. Therefore, to improve safety and eliminate the influencing factors, we should consider and analyze the toxicity, modify the material properties that may harm human health, establish adaptive structures to avoid harmful substances entering blood circulation, and carry out strict in-vitro and in-vivo tests, which is particularly essential for ensuring its wide application [58].

Second, CHs are often composed of many kinds of biomaterials. The composition and proportion of these biomaterials are closely related to safety and effectiveness. Therefore, rigorous research is needed. On the other hand, due to the diversity of conductive materials and hydrogel materials, selecting suitable material combinations becomes perplexing to optimize the biological properties of CHs [80]. Therefore, some conditions (such as biocompatibility, degradability, microstructure, mechanical properties, and conductivity) need to be determined in advance to select suitable materials, further improve and perfect their characteristics, and better simulate ECM to provide accurate control of cellular mechanism.

Third, there are still many unsolved problems regarding the relationship between electrical properties and cell function, so research on the mechanism by which CHs promote peripheral nerve regeneration cannot be ignored. In addition, the study of peripheral nerve development and injury mechanisms may provide useful progress for the treatment of PNI related diseases and peripheral nerve regeneration [81]. Therefore, according to the bioelectric characteristics of PNS, optimizing the biological performance of CHs to increase its adaptability and clarifying the genes and signal pathways regulated by CHs will pave the way for novel CHs development.

Last, most of the ongoing studies are at the preclinical level, so sufficient clinical tests are needed before CHs are put into use to prove that CHs are completely safe for humans and effective for PNIs repair. Accurate operation guidelines should also be developed through repeated trials to guide doctors’ treatment [82]. In addition, unlike animal experiments, we cannot ignore therapeutic evaluation methods that are suitable for humans [83]. At the same time, CHs also have technical problems, such as the structure and effect of CHs may change under the influence of several factors in the human body and then affect the repair effect of CHs.

The above are urgent problems to be solved in the application of CHs. If these problems can be completely solved, CHs are expected to be widely used in clinical practice.

## 4. Conclusions

In this review, we summarized the latest progress of CHs in treating PNIs. CHs have good biocompatibility, mechanical strength, electrical conductivity, and biological activity. CHs are suitable for nerve cells’ survival and are conducive to cell adhesion, infiltration, proliferation, migration, differentiation, and synapse formation. CHs can promote the remyelination of injured axons in PNIs and have great potential to promote nerve regeneration. They show significant advantages and gratifying effects in the symptom control of PNIs and peripheral nerve repair. In future clinical practice, the use of novel CHs to enhance the repair effect of PNIs will have promising prospects in the field of nerve regeneration and tissue engineering. Nevertheless, there is still a certain gap, which needs immediate attention to accelerate clinical transformation. This review provides a useful strategy for neural tissue engineering for PNIs treatment and provides a new idea for clinical treatment.

## Figures and Tables

**Table 1:** Representative examples of electroconductive hydrogels for peripheral nerve regeneration. Abbreviations used in the table are found in the table footer.

Conductive Matrix	In Vitro Studies	In Vivo Studies	Reference
PEDOT + agarose	-	Rat (10 mm peroneal nerve gap)	[47]
PEDOT + chitin + CRGD	RSC-96	Rat (10 mm sciatic nerve gap)	[48]
PANi + cellulose	RSC-96	Rat (5 mm sciatic nerve gap)	[49]
PANi + PAM	Toad Sciatic Nerve, NSC, N2a	Rat (10 mm sciatic nerve gap)	[50]
PPy + alginate + chitosan	BMMSC, RSC-96, PC-12	Rat (10 mm sciatic nerve gap)	[51]
PPy + GelMA + OCS	Rat DRG, PC-12	Rat (diabetic skin wound)	[52]
PPy + TA	Rat DRG, RSC-96, PC-12	Rat (diabetic sciatic nerve crush injury)	[53]
CNT + Matrigel	Rat DRG	-	[54]
CNT + SAP	Rat DRG	-	[55]
reduced (GO/GelMA)	PC-12	Rat (10 mm sciatic nerve gap)	[56]
GO + PPy + alginate	BMMSC	Rat (skin nerves removed)	[57]
Graphene + GelMA + alginate	RSC-96	Rat (10 mm sciatic nerve gap)	[58]

PEDOT = poly(3,4-ethylenedioxythiophene); CRGD = tetrapeptide Cys−Arg−Gly−Asp; PANi = polyaniline; PAM = polyacrylamide; NSC = neural stem cells; PPy = polypyrrole; GelMA = gelatin methacryloyl; OCS = oxidized chondroitin sulfate; DRG = dorsal root ganglion; TA = tannic acid; CNT = carbon nanotube; SAP = self-assembling peptide; GO = graphene oxide; BMMSC = bone marrow mesenchymal stem cell.

## References

[1] Li C., Liu S.Y., Pi W., Zhang P.X. (2021). Cortical plasticity and nerve regeneration after peripheral nerve injury. Neural Regen. Res..

[2] Hewson D.W., Bedforth N.M., Hardman J.G. (2018). Peripheral nerve injury arising in anaesthesia practice. Anaesthesia.

[3] Previtali S.C. (2021). Peripheral Nerve Development and the Pathogenesis of Peripheral Neuropathy: The Sorting Point. Neurotherapeutics.

[4] Nazareth L., St John J., Murtaza M., Ekberg J. (2021). Phagocytosis by Peripheral Glia: Importance for Nervous System Functions and Implications in Injury and Disease. Front. Cell Dev. Biol..

[5] Nocera G., Jacob C. (2020). Mechanisms of Schwann cell plasticity involved in peripheral nerve repair after injury. Cell. Mol. Life Sci..

[6] Li C., Zhang M., Liu S.-Y., Zhang F.-S., Wan T., Ding Z.-T., Zhang P.-X. (2021). Chitin Nerve Conduits with Three-Dimensional Spheroids of Mesenchymal Stem Cells from SD Rats Promote Peripheral Nerve Regeneration. Polymers.

[7] Kaye A.D., Ridgell S., Alpaugh E.S., Mouhaffel A., Kaye A.J., Cornett E.M., Chami A.A., Shah R., Dixon B.M., Viswanath O. (2021). Peripheral Nerve Stimulation: A Review of Techniques and Clinical Efficacy. Pain Ther..

[8] Jha M.K., Passero J.V., Rawat A., Ament X.H., Yang F., Vidensky S., Collins S.L., Horton M.R., Hoke A., Rutter G.A. (2021). Macrophage monocarboxylate transporter 1 promotes peripheral nerve regeneration after injury in mice. J. Clin. Investig..

[9] Fissel J.A., Farah M.H. (2021). The influence of BACE1 on macrophage recruitment and activity in the injured peripheral nerve. J. Neuroinflamm..

[10] Tricaud N., Park H.T. (2017). Wallerian demyelination: Chronicle of a cellular cataclysm. Cell. Mol. Life Sci..

[11] Quintes S., Brinkmann B.G., Ebert M., Frob F., Kungl T., Arlt F.A., Tarabykin V., Huylebroeck D., Meijer D., Suter U. (2016). Zeb2 is essential for Schwann cell differentiation, myelination and nerve repair. Nat. Neurosci..

[12] Min Q., Parkinson D.B., Dun X.P. (2021). Migrating Schwann cells direct axon regeneration within the peripheral nerve bridge. Glia.

[13] Carr M.J., Johnston A.P. (2017). Schwann cells as drivers of tissue repair and regeneration. Curr. Opin. Neurobiol..

[14] Liu S., Liu Y., Zhou L., Li C., Zhang M., Zhang F., Ding Z., Wen Y., Zhang P. (2021). XT-type DNA hydrogels loaded with VEGF and NGF promote peripheral nerve regeneration via a biphasic release profile. Biomater. Sci..

[15] Li R., Li D.H., Zhang H.Y., Wang J., Li X.K., Xiao J. (2020). Growth factors-based therapeutic strategies and their underlying signaling mechanisms for peripheral nerve regeneration. Acta Pharmacol. Sin..

[16] Ino D., Iino M. (2017). Schwann cell mitochondria as key regulators in the development and maintenance of peripheral nerve axons. Cell. Mol. Life Sci..

[17] Wofford K.L., Shultz R.B., Burrell J.C., Cullen D.K. (2021). Neuroimmune interactions and immunoengineering strategies in peripheral nerve repair. Prog. Neurobiol..

[18] Zhang S.H., Shurin G.V., Khosravi H., Kazi R., Kruglov O., Shurin M.R., Bunimovich Y.L. (2020). Immunomodulation by Schwann cells in disease. Cancer Immunol. Immunother..

[19] Vijayavenkataraman S. (2020). Nerve guide conduits for peripheral nerve injury repair: A review on design, materials and fabrication methods. Acta Biomater..

[20] Manoukian O.S., Baker J.T., Rudraiah S., Arul M.R., Vella A.T., Domb A.J., Kumbar S.G. (2020). Functional polymeric nerve guidance conduits and drug delivery strategies for peripheral nerve repair and regeneration. J. Control. Release.

[21] Magaz A., Faroni A., Gough J.E., Reid A.J., Li X., Blaker J.J. (2018). Bioactive Silk-Based Nerve Guidance Conduits for Augmenting Peripheral Nerve Repair. Adv. Healthc. Mater..

[22] Zhang M., Li L., An H., Zhang P., Liu P. (2021). Repair of Peripheral Nerve Injury Using Hydrogels Based on Self-Assembled Peptides. Gels.

[23] Zhang M., Li C., Zhou L.P., Pi W., Zhang P.X. (2021). Polymer Scaffolds for Biomedical Applications in Peripheral Nerve Reconstruction. Molecules.

[24] Koo J., MacEwan M.R., Kang S.K., Won S.M., Stephen M., Gamble P., Xie Z., Yan Y., Chen Y.Y., Shin J. (2018). Wireless bioresorbable electronic system enables sustained nonpharmacological neuroregenerative therapy. Nat. Med..

[25] Carvalho C.R., Silva-Correia J., Oliveira J.M., Reis R.L. (2019). Nanotechnology in peripheral nerve repair and reconstruction. Adv. Drug Deliv. Rev..

[26] Xue W., Shi W., Kong Y., Kuss M., Duan B. (2021). Anisotropic scaffolds for peripheral nerve and spinal cord regeneration. Bioact. Mater..

[27] Dixon A.R., Jariwala S.H., Bilis Z., Loverde J.R., Pasquina P.F., Alvarez L.M. (2018). Bridging the gap in peripheral nerve repair with 3D printed and bioprinted conduits. Biomaterials.

[28] Riccio M., Marchesini A., Pugliese P., De Francesco F. (2019). Nerve repair and regeneration: Biological tubulization limits and future perspectives. J. Cell. Physiol..

[29] Shahriari D., Rosenfeld D., Anikeeva P. (2020). Emerging Frontier of Peripheral Nerve and Organ Interfaces. Neuron.

[30] Manousiouthakis E., Park J., Hardy J.G., Lee J.Y., Schmidt C.E. Towards the translation of electroconductive organic materials for regeneration of neural tissues. Acta Biomater..

[31] Jin F., Li T., Yuan T., Du L., Lai C., Wu Q., Zhao Y., Sun F., Gu L., Wang T. (2021). Physiologically Self-Regulated, Fully Implantable, Battery-Free System for Peripheral Nerve Restoration. Adv. Mater..

[32] Yao X., Qian Y., Fan C. (2021). Electroactive nanomaterials in the peripheral nerve regeneration. J. Mater. Chem. B.

[33] Seo H., Han S.I., Song K.I., Seong D., Lee K., Kim S.H., Park T., Koo J.H., Shin M., Baac H.W. (2021). Durable and Fatigue-Resistant Soft Peripheral Neuroprosthetics for In Vivo Bidirectional Signaling. Adv. Mater..

[34] Liu Y., Liu J., Chen S., Lei T., Kim Y., Niu S., Wang H., Wang X., Foudeh A.M., Tok J.B. (2019). Soft and elastic hydrogel-based microelectronics for localized low-voltage neuromodulation. Nat. Biomed. Eng..

[35] Piantanida E., Alonci G., Bertucci A., De Cola L. (2019). Design of Nanocomposite Injectable Hydrogels for Minimally Invasive Surgery. Acc. Chem. Res..

[36] Porzionato A., Barbon S., Stocco E., Dalzoppo D., Contran M., De Rose E., Parnigotto P.P., Macchi V., Grandi C., De Caro R. (2019). Development of Oxidized Polyvinyl Alcohol-Based Nerve Conduits Coupled with the Ciliary Neurotrophic Factor. Materials.

[37] Ye L., Ji H., Liu J., Tu C.H., Kappl M., Koynov K., Vogt J., Butt H.J. (2021). Carbon Nanotube-Hydrogel Composites Facilitate Neuronal Differentiation While Maintaining Homeostasis of Network Activity. Adv. Mater..

[38] De Lima G.G., Junior E.L.S., Aggio B.B., Shee B.S., Filho E.M.M., Segundo F.A.S., Fournet M.B., Devine D.M., Magalhaes W.L.E., de Sa M.J.C. (2021). Nanocellulose for peripheral nerve regeneration in rabbits using citric acid as crosslinker with chitosan and freeze/thawed PVA. Biomed. Mater..

[39] Hull S.M., Brunel L.G., Heilshorn S.C. (2021). 3D Bioprinting of Cell-Laden Hydrogels for Improved Biological Functionality. Adv. Mater..

[40] Kapoor S., Kundu S.C. (2016). Silk protein-based hydrogels: Promising advanced materials for biomedical applications. Acta Biomater..

[41] Samadian H., Maleki H., Fathollahi A., Salehi M., Gholizadeh S., Derakhshankhah H., Allahyari Z., Jaymand M. (2020). Naturally occurring biological macromolecules-based hydrogels: Potential biomaterials for peripheral nerve regeneration. Int. J. Biol. Macromol..

[42] Meder T., Prest T., Skillen C., Marchal L., Yupanqui V.T., Soletti L., Gardner P., Cheetham J., Brown B.N. (2021). Nerve-specific extracellular matrix hydrogel promotes functional regeneration following nerve gap injury. NPJ Regen. Med..

[43] Guiseppi-Elie A. (2010). Electroconductive hydrogels: Synthesis, characterization and biomedical applications. Biomaterials.

[44] Walker B.W., Lara R.P., Mogadam E., Yu C.H., Kimball W., Annabi N. (2019). Rational Design of Microfabricated Electroconductive Hydrogels for Biomedical Applications. Prog. Polym. Sci..

[45] Liang S., Zhang Y., Wang H., Xu Z., Chen J., Bao R., Tan B., Cui Y., Fan G., Wang W. (2018). Paintable and Rapidly Bondable Conductive Hydrogels as Therapeutic Cardiac Patches. Adv. Mater..

[46] Nezakati T., Seifalian A., Tan A., Seifalian A.M. (2018). Conductive Polymers: Opportunities and Challenges in Biomedical Applications. Chem. Rev..

[47] Abidian M.R., Daneshvar E.D., Egeland B.M., Kipke D.R., Cederna P.S., Urbanchek M.G. (2012). Hybrid conducting polymer-hydrogel conduits for axonal growth and neural tissue engineering. Adv. Healthc. Mater..

[48] Huang L., Yang X., Deng L., Ying D., Lu A., Zhang L., Yu A., Duan B. (2021). Biocompatible Chitin Hydrogel Incorporated with PEDOT Nanoparticles for Peripheral Nerve Repair. ACS Appl. Mater. Interfaces.

[49] Xu D.F., Fan L., Gao L.F., Xiong Y., Wang Y.F., Ye Q.F., Yu A.X., Dai H.L., Yin Y.X., Cai J. (2016). Micro-Nanostructured Polyaniline Assembled in Cellulose Matrix via Interfacial Polymerization for Applications in Nerve Regeneration. ACS Appl. Mater. Interfaces.

[50] Dong M., Shi B., Liu D., Liu J.H., Zhao D., Yu Z.H., Shen X.Q., Gan J.M., Shi B.L., Qiu Y. (2020). Conductive Hydrogel for a Photothermal-Responsive Stretchable Artificial Nerve and Coalescing with a Damaged Peripheral Nerve. ACS Nano.

[51] Bu Y., Xu H.X., Li X., Xu W.J., Yin Y.X., Dai H.L., Wang X.B., Huang Z.J., Xu P.H. (2018). A conductive sodium alginate and carboxymethyl chitosan hydrogel doped with polypyrrole for peripheral nerve regeneration. Rsc. Adv..

[52] Fan L., Xiao C., Guan P., Zou Y., Wen H., Liu C., Luo Y., Tan G., Wang Q., Li Y. (2021). Extracellular Matrix-Based Conductive Interpenetrating Network Hydrogels with Enhanced Neurovascular Regeneration Properties for Diabetic Wounds Repair. Adv. Healthc. Mater..

[53] Liu C., Fan L., Tian Z.M., Wen H.Q., Zhou L., Guan P.F., Luo Y., Chan C.C., Tan G.X., Ning C.Y. (2021). Self-curling electroconductive nerve dressing for enhancing peripheral nerve regeneration in diabetic rats. Bioact. Mater..

[54] Koppes A.N., Keating K.W., McGregor A.L., Koppes R.A., Kearns K.R., Ziemba A.M., McKay C.A., Zuidema J.M., Rivet C.J., Gilbert R.J. (2016). Robust neurite extension following exogenous electrical stimulation within single walled carbon nanotube-composite hydrogels. Acta Biomater..

[55] He L.M., Xiao Q., Zhao Y.Y., Li J., Reddy S., Shi X.S., Su X., Chiu K., Ramakrishna S. (2020). Engineering an Injectable Electroactive Nanohybrid Hydrogel for Boosting Peripheral Nerve Growth and Myelination in Combination with Electrical Stimulation. Acs Appl. Mater. Interfaces.

[56] Park J., Jeon J., Kim B., Lee M.S., Park S., Lim J., Yi J., Lee H., Yang H.S., Lee J.Y. (2020). Electrically Conductive Hydrogel Nerve Guidance Conduits for Peripheral Nerve Regeneration. Adv. Funct. Mater..

[57] Peng L.H., Xu X.H., Huang Y.F., Zhao X.L., Zhao B., Cai S.Y., Xie M.J., Wang M.Z., Yuan T.J., He Y. (2020). Self-Adaptive All-In-One Delivery Chip for Rapid Skin Nerves Regeneration by Endogenous Mesenchymal Stem Cells. Adv. Funct. Mater..

[58] Huang Q., Cai Y.T., Zhang X., Liu J.C., Liu Z.J., Li B., Wong H.L., Xu F., Sheng L.Y., Sun D.Z. (2021). Aligned Graphene Mesh-Supported Double Network Natural Hydrogel Conduit Loaded with Netrin-1 for Peripheral Nerve Regeneration. ACS Appl. Mater. Interfaces.

[59] Luo H., Kaneti Y.V., Ai Y., Wu Y., Wei F., Fu J., Cheng J., Jing C., Yuliarto B., Eguchi M. (2021). Nanoarchitectured Porous Conducting Polymers: From Controlled Synthesis to Advanced Applications. Adv. Mater..

[60] Lee S., Ozlu B., Eom T., Martin D.C., Shim B.S. (2020). Electrically conducting polymers for bio-interfacing electronics: From neural and cardiac interfaces to bone and artificial tissue biomaterials. Biosens. Bioelectron..

[61] Balint R., Cassidy N.J., Cartmell S.H. (2014). Conductive polymers: Towards a smart biomaterial for tissue engineering. Acta Biomater..

[62] Tandon B., Magaz A., Balint R., Blaker J.J., Cartmell S.H. (2018). Electroactive biomaterials: Vehicles for controlled delivery of therapeutic agents for drug delivery and tissue regeneration. Adv. Drug Deliv. Rev..

[63] Talikowska M., Fu X., Lisak G. (2019). Application of conducting polymers to wound care and skin tissue engineering: A review. Biosens. Bioelectron..

[64] Kayser L.V., Lipomi D.J. (2019). Stretchable Conductive Polymers and Composites Based on PEDOT and PEDOT:PSS. Adv. Mater..

[65] Manjakkal L., Pullanchiyodan A., Yogeswaran N., Hosseini E.S., Dahiya R. (2020). A Wearable Supercapacitor Based on Conductive PEDOT:PSS-Coated Cloth and a Sweat Electrolyte. Adv. Mater..

[66] Horev Y.D., Maity A., Zheng Y., Milyutin Y., Khatib M., Yuan M., Suckeveriene R.Y., Tang N., Wu W., Haick H. (2021). Stretchable and Highly Permeable Nanofibrous Sensors for Detecting Complex Human Body Motion. Adv. Mater..

[67] Zarrintaj P., Bakhshandeh B., Rezaeian I., Heshmatian B., Ganjali M.R. (2017). A Novel Electroactive Agarose-Aniline Pentamer Platform as a Potential Candidate for Neural Tissue Engineering. Sci. Rep..

[68] Xu B., Bai T., Sinclair A., Wang W., Wu Q., Gao F., Jia H., Jiang S., Liu W. (2016). Directed neural stem cell differentiation on polyaniline-coated high strength hydrogels. Mater. Today Chem..

[69] Bao B., Rivkin B., Akbar F., Karnaushenko D.D., Bandari V.K., Teuerle L., Becker C., Baunack S., Karnaushenko D., Schmidt O.G. (2021). Digital Electrochemistry for On-Chip Heterogeneous Material Integration. Adv. Mater..

[70] Zhao Y., Liang Y., Ding S., Zhang K., Mao H.Q., Yang Y. (2020). Application of conductive PPy/SF composite scaffold and electrical stimulation for neural tissue engineering. Biomaterials.

[71] Li F., Wang R., Song C., Zhao M., Ren H., Wang S., Liang K., Li D., Ma X., Zhu B. (2021). A Skin-Inspired Artificial Mechanoreceptor for Tactile Enhancement and Integration. ACS Nano.

[72] Kinloch I.A., Suhr J., Lou J., Young R.J., Ajayan P.M. (2018). Composites with carbon nanotubes and graphene: An outlook. Science.

[73] Chen M., Qin X., Zeng G. (2017). Biodegradation of Carbon Nanotubes, Graphene, and Their Derivatives. Trends Biotechnol..

[74] Jordan J.W., Townsend W.J.V., Johnson L.R., Walsh D.A., Newton G.N., Khlobystov A.N. (2021). Electrochemistry of redox-active molecules confined within narrow carbon nanotubes. Chem. Soc. Rev..

[75] Sun X., Huang C., Wang L., Liang L., Cheng Y., Fei W., Li Y. (2021). Recent Progress in Graphene/Polymer Nanocomposites. Adv. Mater..

[76] Wang J., Wang H., Mo X., Wang H. (2020). Reduced Graphene Oxide-Encapsulated Microfiber Patterns Enable Controllable Formation of Neuronal-Like Networks. Adv. Mater..

[77] Li G., Zheng T., Wu L., Han Q., Lei Y., Xue L., Zhang L., Gu X., Yang Y. (2021). Bionic microenvironment-inspired synergistic effect of anisotropic micro-nanocomposite topology and biology cues on peripheral nerve regeneration. Sci. Adv..

[78] Mobini S., Song Y.H., McCrary M.W., Schmidt C.E. (2019). Advances in ex vivo models and lab-on-a-chip devices for neural tissue engineering. Biomaterials.

[79] Du J., Chen H., Qing L., Yang X., Jia X. (2018). Biomimetic neural scaffolds: A crucial step towards optimal peripheral nerve regeneration. Biomater. Sci..

[80] Lu H., Zhang N., Ma M. (2019). Electroconductive hydrogels for biomedical applications. Wiley Interdiscip. Rev. Nanomed. Nanobiotechnol..

[81] Fledrich R., Kungl T., Nave K.A., Stassart R.M. (2019). Axo-glial interdependence in peripheral nerve development. Development.

[82] Moskow J., Ferrigno B., Mistry N., Jaiswal D., Bulsara K., Rudraiah S., Kumbar S.G. (2019). Review: Bioengineering approach for the repair and regeneration of peripheral nerve. Bioact. Mater..

[83] Wieringa P.A., Goncalves de Pinho A.R., Micera S., van Wezel R.J.A., Moroni L. (2018). Biomimetic Architectures for Peripheral Nerve Repair: A Review of Biofabrication Strategies. Adv. Healthc. Mater..

